# Association between hemoglobin variability and mortality in hemodialysis patients: a retrospective cohort study

**DOI:** 10.3389/fmed.2026.1707546

**Published:** 2026-03-11

**Authors:** Zhizhen Zhou, Feng Wang, Sisi Lin, Dieyu Ma, Conghua Ji

**Affiliations:** 1 School of Public Health, Zhejiang Chinese Medical University, Hangzhou, China; 2 Department of Nephrology, Wenzhou TCM Hospital of Zhejiang Chinese Medical University, Wenzhou, China

**Keywords:** coefficient of variation, hemodialysis, hemoglobin, mortality, prognosis

## Abstract

**Background:**

Patients undergoing hemodialysis (HD) face high mortality rates. Monitoring specific laboratory parameters and their derivatives, including hemoglobin (Hb) and its variability, is crucial for improving clinical management and patient outcomes. However, the association between hemoglobin variability and mortality in HD patients remains controversial.

**Methods:**

A total of 652 HD patients treated at Wenzhou Traditional Chinese Medicine Hospital of Zhejiang Chinese Medical University were included based on predefined criteria. Patients were divided into survival group and death group based on mortality status during the study period for univariate analysis. Based on univariate results, the following parameters representing Hb variability (Hb-Var) were studied: Hb standard deviation (SD), Hb residual SD, Hb coefficient of variation (CV), and Hb abnormal ratio. Cut-off values for each parameter were determined using ROC curves. Patients were stratified into high-risk group and low-risk group based on these cut-off values. Survival analysis was performed using multivariable Cox regression to obtain hazard ratios (HRs) and *p*-values for each parameter. To ensure robustness, we repeated the same analyses in the subgroup of patients with at least 10 CBC tests.

**Results:**

The results of univariate analysis showed that there were statistically significant differences in age (*p* < 0.001), mean Hb (*p* = 0.002), Hb CV (*p* = 0.007), Hb residual SD (*p* < 0.001), and Hb abnormal ratio (*p* = 0.001). ROC curve analysis yielded the following AUC values: Mean Hb HR = 0.37 (95% CI: 0.23–0.59, *p* < 0.001), Hb SD HR = 1.72 (95% CI: 1.09–2.72, *p* = 0.019), Hb residual SD HR = 1.87 (95% CI: 1.20–2.91, *p* = 0.006), Hb CV HR = 2.12 (95% CI: 1.34–3.35, *p* = 0.001), Hb abnormal ratio HR = 2.30 (95% CI:1.43–3.69, *p* < 0.001). The results of the subgroup were largely similar.

**Conclusion:**

Hb-Var included Hb abnormal ratio, Hb residual SD and Hb CV are independent factors associated with mortality in hemodialysis patients.

## 1 Introduction

Patients undergoing hemodialysis (HD) experience high mortality, and this high mortality has not improved much in the past decade. On the contrary, it may increase due to some emergencies (such as communicable diseases pandemic). For example, according to the 2024 Annual Data Report [The analyses conducted for this chapter use data from the Centers for Medicare & Medicaid Services (CMS), the End Stage Renal Disease Quality Reporting System (EQRS), and the Organ Procurement and Transplantation Network (OPTN)] from the United States Renal Data System (USRDS), the adjusted mortality among US HD patients was 179.4 per 1,000 patient-years in 2012 and 166.0 per 1,000 patient-years in 2019, representing a 7.5% decline. Because of COVID-19 pandemic, mortality rose to 194.4 per 1,000 patient-years in 2020, then it decreased to 185.1 per 1,000 patient-years in 2022 (1). This persistent high mortality rate underscores the need for improved prognostic tools and management strategies to enhance survival outcomes in this vulnerable population.

A major contributor to this high mortality is the high prevalence of complications in HD patients, such as anemia, malnutrition, and vascular calcification. These complications are intricately linked to a persistent inflammatory state underlying adverse outcomes, finally leading to increased frailty, risk of infections and mortality (2). The complex interplay between these factors creates a challenging clinical environment where patients are at continuous risk of deteriorating health. Understanding and monitoring these complications through reliable biomarkers is essential for timely intervention and improved patient care.

Generally, anemia is defined as hemoglobin levels below 13 g/dL (130 g/L) in males and below 12 g/dL (120 g/L) in females (3). Hemoglobin levels typically decline alongside decreasing renal function in CKD patients, making anemia highly prevalent in the HD population (4, 5). A study in China reports anemia prevalence as high as 98.2% in dialysis patients (6). Anemia in CKD and HD patients is associated with increased mortality and hospitalization rates (7, 8), warranting significant clinical attention for its monitoring.

The management of anemia in HD patients primarily revolves around the use of erythropoiesis-stimulating agents (ESAs) and iron supplementation, guided by regular monitoring of hemoglobin levels. International clinical guidelines, such as those from the Kidney Disease: Improving Global Outcomes (KDIGO) initiative, recommend targeting a hemoglobin concentration within a specific range (typically 10–11.5 g/dL) to avoid the risks associated with both anemia and high hemoglobin levels (4, 9). While numerous laboratory parameters can monitor anemia, complete blood count (CBC), which includes the core anemia parameter, hemoglobin, is an inexpensive, simple, and widely used laboratory test for anemia monitoring in HD patients (9).

In addition to the studies that directly use specific laboratory test results as predictors of mortality risk in HD patients, there are many studies focusing on the predictive ability of the variability of these laboratory test results on the mortality risk of HD patients. Concurrently, some studies have demonstrated associations between mortality in HD patients and variability (often represented by CV) of systolic blood pressure (10), blood glucose in diabetic patients (11), serum potassium (12), serum sodium (13), and serum magnesium (14). This highlights the potential of laboratory parameter variability, particularly CV, in predicting mortality risk in HD patients.

At the same time, some previous studies have explored the association between Hb variability and mortality in HD patients (15–17), these studies primarily using Hb SD and residual SD to represent variability. However, their findings are incongruent. Some studies, utilizing parameters like standard deviation (SD) and residual SD, have reported a significant association (15, 16), while others, employing similar parameters, have found no independent link (17). Specifically, Brunelli et al.’s (15) study found that, in the primary analysis, each g/dl increase in hemoglobin variability was associated with an adjusted hazard ratio (HR) [95% confidence interval (CI)] for all-cause mortality of 1.93 (1.20 to 3.10). Neither higher absolute hemoglobin level nor increasing hemoglobin trend were significantly associated with mortality; adjusted HR (95% CI) 0.85 (0.64 to 1.11) and 0.60 (0.25 to 1.45), respectively. Meanwhile, In Eckardt et al.’s (17) study, multivariable adjusted Cox regression showed that Hb’s SD, residual SD, time-in-target, and AUC did not predict all-cause or CVD mortality during a median follow-up of 12.4 months (IQR: 7.7 to 17.4). Only such results can be confirmed: patients with consistently low levels of Hb (<11 g/dL) and those who fluctuated between the target range and <11 g/dL had increased risks for death (RR 2.34; 95% CI: 1.24 to 4.41 and RR 1.74; 95% CI: 1.00 to 3.04, respectively). This discrepancy may stem from differences in study populations, statistical methodologies, and the specific variability parameters chosen. Crucially, the coefficient of variation (CV)—a dimensionless measure that normalizes variability to the mean, allowing for better comparison between individuals—has been scarcely investigated. Furthermore, the clinically intuitive concept of the “abnormal ratio” has not been evaluated as a marker of Hb-Var. Therefore, a comprehensive evaluation incorporating these diverse parameters is warranted to clarify the true prognostic value of Hb-Var. Moreover, studies investigating the Hb abnormal ratio are lacking in literature.

Our study evaluates the association between Hb variability and mortality in incident HD patients, represented by Hb SD, Hb residual SD, Hb CV, and the Hb abnormal ratio. By including these four parameters, we aim to provide a comprehensive analysis of Hb-Var and its relationship with mortality, addressing gaps in the existing literature and offering insights into the most effective metrics for risk stratification.

## 2 Methods

### 2.1 Study design

This is a retrospective cohort study. This study has been approved by the Ethics Committee of Wenzhou Traditional Chinese medicine Hospital of Zhejiang Chinese Medical University (No. WZY2025-LW-020-01). Due to the retrospective analysis of historical data in this study, the ethics committee exempted participants from informed consent.

### 2.2 Inclusion criteria

The detailed inclusion criteria include: (1) Hospitalized patients; (2) Receiving hemodialysis treatment at the Wenzhou Traditional Chinese medicine Hospital of Zhejiang Chinese Medical University (Hereinafter referred to as Wenzhou TCM Hospital) between October 30, 2017, and September 21, 2024; (3) Aged 18 years or older; (4) Having at least three CBC test results (to assess the impact of data density, same analyses were also performed on the subgroup with at least 10 CBC test results).

### 2.3 Exclusion criteria

(1) Under 18 years old; (2) Fewer than 3 CBC tests; (3) Incomplete or missing key data, such as age, dialysis initiation date, or date of death.

### 2.4 Participants

Between October 30, 2017, and September 21, 2024, 1,217 hospitalized patients received at least one HD session at Wenzhou TCM Hospital. All patients are aged 18 years or older. All laboratory data were extracted from the hospital’s electronic medical record system.

### 2.5 Hemoglobin variability parameters

Hemoglobin variability (Hb-Var) was represented by four parameters:

Hemoglobin standard deviation (SD): The standard deviation of all Hb measurements for an individual patient, calculated as:


$$s=\sqrt{\frac{1}{n-1}\sum_{i=1}^{n}{\left(x_{i}-\bar{x}\right)}^{2}}$$

Where *n* is the total number of tests for the patient, *xᵢ* is the value of a single test result of that patient, and *x̄* is the mean of all test results for the same patient. This parameter provides a measure of the absolute variability in Hb levels, capturing the dispersion of measurements around the mean.

Hemoglobin residual standard deviation (Residual SD): Obtained by fitting a time-based linear model (using ordinary least squares) to each patient’s Hb measurements over time. The standard deviation of the distances (residuals) between each individual Hb measurement and the best-fit line is the residual SD (10). This parameter accounts for the trend in Hb levels over time, providing a measure of variability that is independent of any linear change.

Coefficient of variation (CV): Calculated as:


$$\mathrm{CV}=\frac{s}{\bar{x}}\times 100\%$$

Where *s* is the standard deviation of all test results for a specific parameter in one patient, and *x̄* is the mean of all test results for that parameter in the same patient. The CV normalizes variability to the mean, allowing for comparison between patients with different average Hb levels.

Abnormal ratio values: Calculated as the total number of times a laboratory parameter was outside the normal range divided by the total number of tests for that parameter, then multiplied by 100%. This parameter provides a clinically intuitive measure of how often Hb levels deviate from the target range, reflecting the proportion of measurements that are outside the desired clinical parameters.

### 2.6 Demographic and clinical data

All baseline data, including demographic details (age, gender) and laboratory results, were collected at HD initiation. In our study, the normal range for hemoglobin was 130–175 g/L for males and 115–150 g/L for females.

### 2.7 Outcome

The primary outcome was all-cause mortality. Survival time was defined as the period from enrollment until death or administrative censoring.

### 2.8 Statistical analysis

Continuous variables are expressed as mean±standard deviation (SD) unless otherwise specified. Comparisons between parameters were made using paired or independent *t*-tests, *χ*^2^ tests, or Wilcoxon rank-sum tests as appropriate. Pearson’s correlation coefficient was used for normally distributed variables. Patients were stratified into survival group and death group for univariate analysis based on mortality status. Cut-off values for study factors were determined using ROC curves. Patients were stratified into high-risk group and low-risk group based on these cut-off values. Conduct survival analysis based on the above grouping. Survival analysis was performed using multivariable Cox regression to obtain HRs and *p*-values for each study factor. Statistical significance for all analyses was defined as a two-sided *p* value < 0.05. The above conditions apply to the entire cohort and the subgroup. SPSS 27.0 (IBM Corp., Armonk, NY, USA) and R 4.3.3 (R Foundation for Statistical Computing, Vienna, Austria) were used for statistical analyses.

## 3 Results

### 3.1 Baseline characters

Ultimately, 652 patients who received at least one HD treatment during this period were included. Their mean age was 69.5 ± 13.6 years, with a median age of 70 (23, 101) years. These patients included 412 males (63.2%). The average first hemoglobin level of all patients was 93.37 ± 20.86 g/L. All patients underwent a total of 28,398 CBC tests, while each patient underwent 43.56 (±36.98) tests in average. One of these patients had the highest number of tests (163 CBC tests) during the study period.

**Figure 1:**
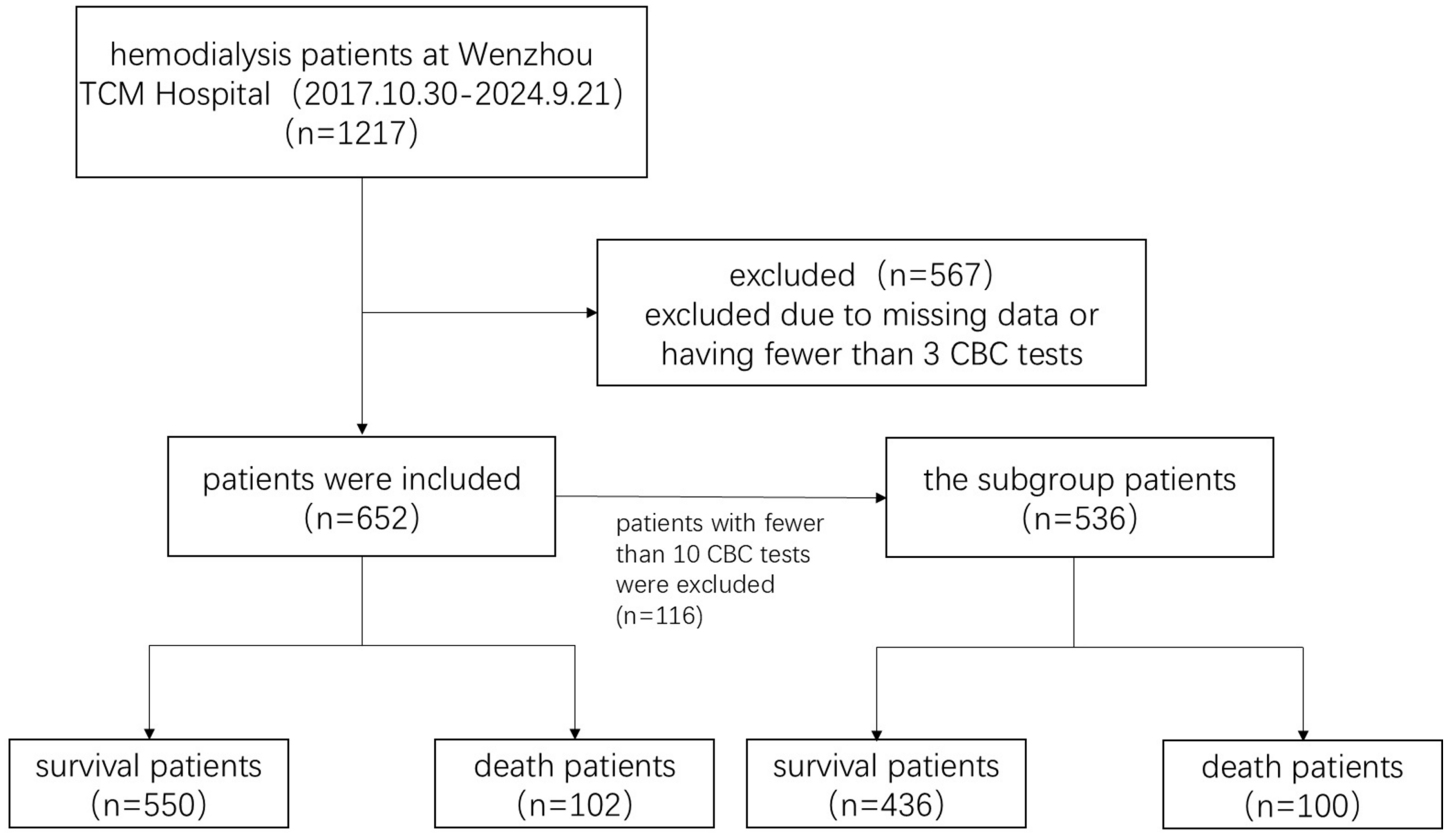
Flow chart for screening subjects.

During the study period, 102 patients died (15.6%) (Figure 1), 62 (60.8%) were male and 40 (39.2%) were female. Among them, 36 (35.3%) died of cardiovascular disease, 19 (18.6%) died of infection, 17 (16.7%) died of multiple organ failure, 15 (14.7%) died of respiratory failure, 9 (8.8%) died of tumors, 1 (1.0%) died of hepatic failure, 1 (1.0%) died of hemorrhage and 4 (3.9%) died of unknown causes. The causes of death partly reflect the common complications associated with HD, highlighting the multifactorial nature of mortality in this population.

The subgroup includes 536 patients. Their mean age was 68.9 ± 13.8 years, with a median age of 70 (23, 101) years. These patients included 345 males (64.4%). The average first hemoglobin level of all patients was 93.11 ± 20.70 g/L. All patients underwent a total of 27,629 CBC tests, while each patient underwent 51.55 (±36.11) tests in average. One of these patients had the highest number of tests (163 CBC tests) during the study period. During the study period, 100 patients in the subgroup died, 62 (62.0%) were male and 38 (38.0%) were female. Among them, 36 (36.0%) died of cardiovascular disease, 18 (18.0%) died of infection, 16 (16.0%) died of multiple organ failure, 15 (15.0%) died of respiratory failure, 9 (9.0%) died of tumors, 1 (1.0%) died of hepatic failure, 1 (1.0%) died of hemorrhage and 4 (4.0%) died of unknown causes.

We conducted a baseline comparison between the entire cohort and the subgroup, which showed no significant differences in age, gender, the first hemoglobin test result, or survival status (Table 1).

**Table 1:** Baseline comparison between the entire cohort and the subgroup.

Variable	Entire cohort (*n* = 652)	Subgroup (*n* = 536)	*p* value
Age	69.50 ± 13.59	68.94 ± 13.80	0.487
First Hb result	93.37 ± 20.86	93.11 ± 20.70	0.833
Gender: Male	412	345	0.720
Female	240	191	
Status: Survival	550	436	0.169
Death	102	100	

Considering the potential indication bias, we used the Wilcoxon rank-sum test to compare the average total number of tests between patients in the survival group and those in the death group, both in the entire cohort and the subgroup. Both results demonstrated a significant difference between the two groups (the entire cohort’s result is *p* < 0.001, the subgroup’s result is *p* = 0.033). Therefore, in subsequent survival analyses, the total number of tests was included as a covariate to adjust for the bias it may introduce.

### 3.2 Univariate analysis based on death status grouping

Univariate analysis comparing the survival (*n* = 550) group and death (*n* = 102) group of the entire cohort. There were statistically significant differences in age (*p* < 0.001), mean Hb (*p* = 0.002), Hb CV (*p* = 0.007), Hb residual SD (*p* < 0.001), and Hb abnormal ratio (*p* = 0.001). There was no significant difference in the Hb SD between two groups. Thus, as shown in Table 2, patients in the death group were significantly older, their mean hemoglobin levels were lower, and their mean hemoglobin coefficient of variation, mean residual SD, and mean Hb abnormal ratio were higher. These findings suggest that both lower average Hb levels and higher Hb variability are associated with increased mortality, supporting the potential prognostic value of Hb-Var.

**Table 2:** Univariate analysis results.

Variable	Survival group (*n* = 550)	Death group (*n* = 102)	*p* value
Age	68.64 ± 13.77	74.14 ± 11.60	<0.001
Gender: Male	350	62	0.662
Female	200	40	
Hb mean (g/L)	98.33 ± 14.51	93.75 ± 13.12	0.002
Hb SD (g/L)	14.01 ± 5.01	14.74 ± 4.33	0.129
Hb CV (%)	14.40 ± 5.34	15.83 ± 4.72	0.007
Hb residual SD (g/L)	11.94 ± 4.67	13.66 ± 4.16	<0.001
Hb abnormal ratio (%)	74.52 ± 21.36	80.69 ± 16.60	0.001

### 3.3 ROC curves and calculating cut-off values

**Figure 2:**
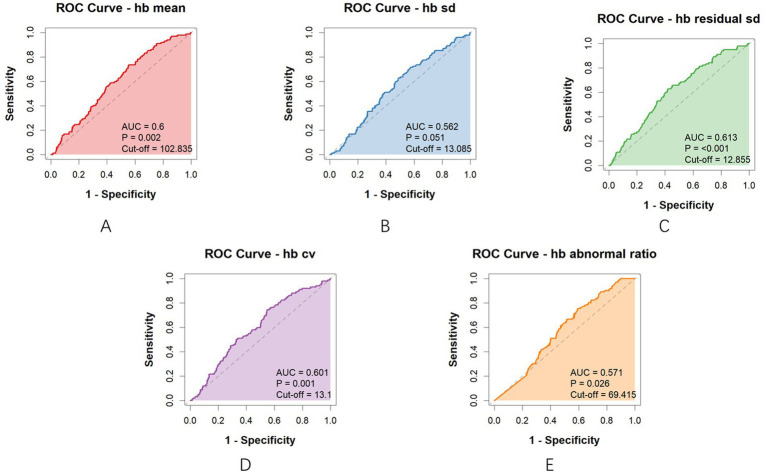
ROC curves and AUC values for each parameter (the entire cohort). **(A)** AUC value and Cut-off of hb mean; **(B)** AUC value and Cut-off of hb sd; **(C)** AUC value and Cut-off of hb residual sd; **(D)** AUC value and Cut-off of hb cv; **(E)** AUC value and Cut-off of hb abnormal ratio.

ROC curve analysis for the entire cohort yielded the following AUC values: Mean Hb AUC = 0.600 (*p* = 0.002), Hb SD AUC = 0.562 (*p* = 0.051), Hb residual SD AUC = 0.613 (*p* < 0.001), Hb CV AUC = 0.601 (*p* = 0.001), Hb Abnormal ratio AUC = 0.571 (*p* = 0.026). Cut-off values for each parameter were also determined (Figure 2).

**Figure 3:**
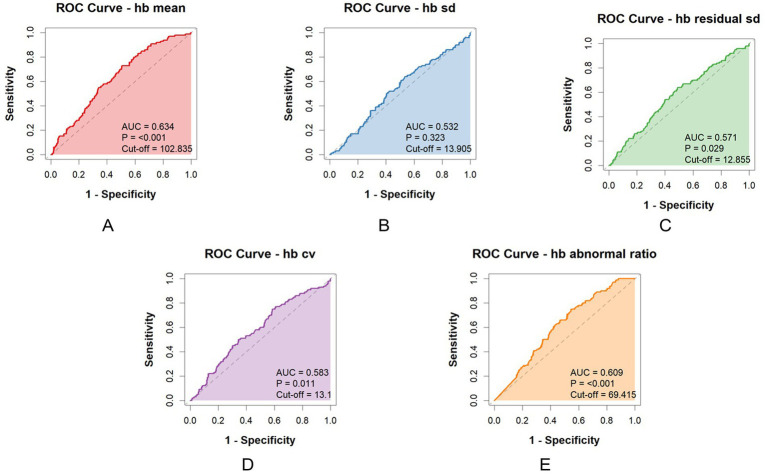
ROC curves and AUC values for each parameter (the subgroup). **(A)** AUC value and Cut-off of hb mean; **(B)** AUC value and Cut-off of hb sd; **(C)** AUC value and Cut-off of hb residual sd; **(D)** AUC value and Cut-off of hb cv; **(E)** AUC value and Cut-off of hb abnormal ratio.

The AUC values of the subgroup are presented below: Mean Hb AUC = 0.634 (*p* < 0.001), Hb SD AUC = 0.532 (*p* = 0.323), Hb residual SD AUC = 0.571 (*p* = 0.029), Hb CV AUC = 0.583 (*p* = 0.011), Hb Abnormal ratio AUC = 0.609 (*p* < 0.001). Cut-off values for each parameter were also determined (Figure 3).

The AUC values indicate moderate predictive accuracy for most parameters, with abnormal ratio, mean, residual SD and CV showing the higher values. The cut-off values provide clinically useful thresholds for risk stratification.

### 3.4 Hb variability and prognosis in the entire cohort and the subgroup (survival analysis)

**Figure 4:**
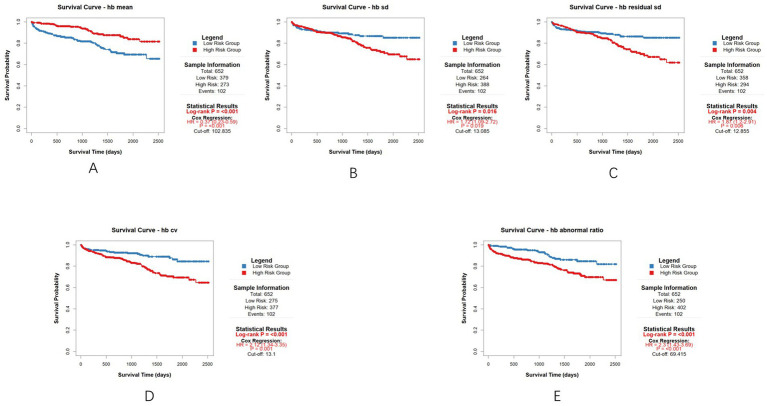
Multivariable Cox regression according to the cut-off value for each parameter (the entire cohort). **(A)** Survival analysis result of hb mean; **(B)** Survival analysis result of hb sd; **(C)** Survival analysis result of hb residual sd; **(D)** Survival analysis result of hb cv); **(E)** Survival analysis result of hb abnormal ratio.

**Figure 5:**
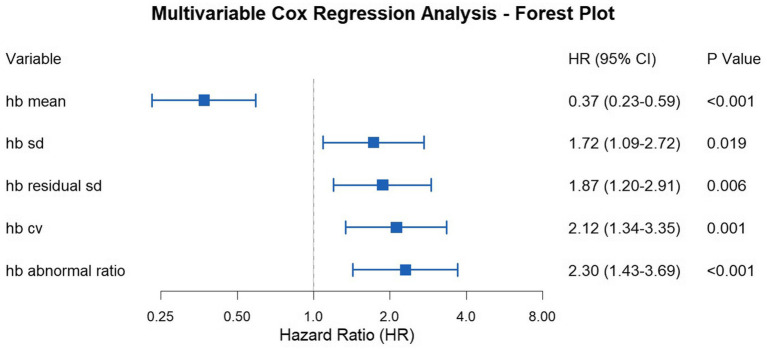
Forest plot of hazard ratios for hemoglobin parameters (the entire cohort).

Patients were stratified into high-risk and low-risk groups based on whether their mean value exceeded the cut-off value. Perform survival analysis based on the above grouping. We performed survival analysis using multivariable Cox regression, adjusted for age, gender, and the total number of tests. We performed the survival analysis for the entire cohort first. Survival analysis results were as follows: Mean Hb HR = 0.37 (95% CI: 0.23–0.59, *p* < 0.001), Hb SD HR = 1.72 (95% CI: 1.09–2.72, *p* = 0.019), Hb residual SD HR = 1.87 (95% CI: 1.20–2.91, *p* = 0.006), Hb CV HR = 2.12 (95% CI: 1.34–3.35, *p* = 0.001), Hb abnormal ratio HR = 2.30 (95% CI:1.43–3.69, *p* < 0.001) (Figure 4). The survival analysis results are summarized in a forest plot (Figure 5).

**Figure 6:**
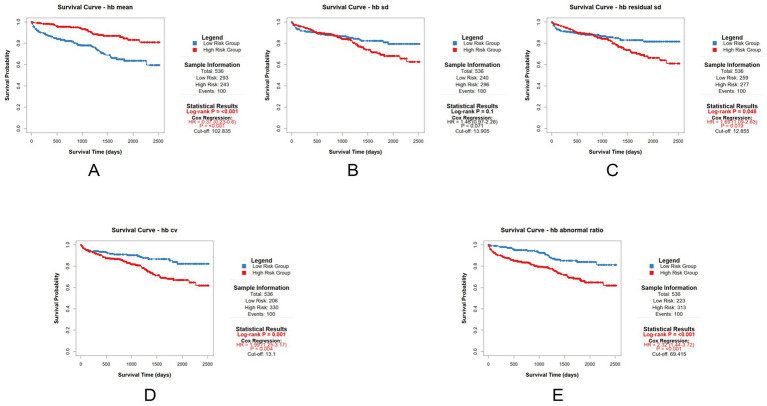
Multivariable Cox regression according to the cut-off value for each parameter (the subgroup). **(A)** Survival analysis result of hb mean; **(B)** Survival analysis result of hb sd; **(C)** Survival analysis result of hb residual sd; **(D)** Survival analysis result of hb cv; **(E)** Survival analysis result of hb abnormal ratio.

**Figure 7:**
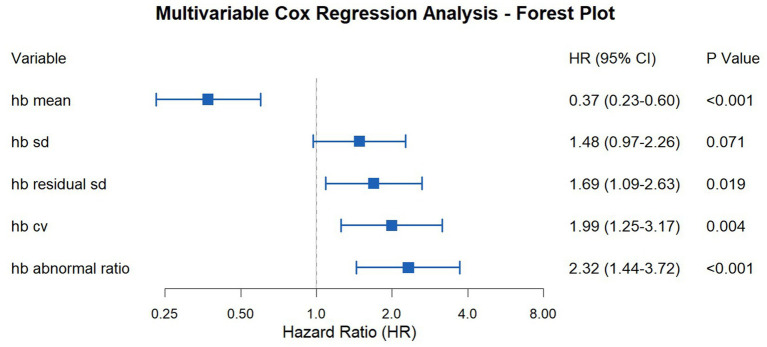
Forest plot of hazard ratios for hemoglobin parameters (the subgroup).

We performed the same survival analysis for the subgroup as for the entire cohort. The survival analysis results for the subgroup are presented below: Mean Hb HR = 0.37 (95% CI: 0.23–0.60, *p* < 0.001), Hb SD HR = 1.48 (95% CI: 0.97–2.26, *p* = 0.071), Hb residual SD HR = 1.69 (95% CI: 1.09–2.63, *p* = 0.019), Hb CV HR = 1.99 (95% CI: 1.25–3.17, *p* = 0.004), Hb abnormal ratio HR = 2.32 (95% CI:1.44–3.72, *p* < 0.001) (Figure 6). These survival analysis results are also summarized in another forest plot (Figure 7).

Based on these results, it can be concluded that hemodialysis patients with Mean Hb, Hb residual SD, Hb CV, and Hb abnormal ratio values above the cut-off value have a higher risk of mortality. The significant HRs for most variability parameters confirm their association with increased mortality, supporting their use as prognostic markers.

## 4 Discussion

This study is the first to analyze the relationship between Hb variability and mortality in HD patients using CV and abnormal ratio, and this study involving 652 HD patients in Wenzhou, China. To ensure robustness, we repeated the same analyses in the subset of patients with at least 10 CBC tests, which yielded comparable results. Consistent with previous studies, our results confirm that Hb abnormal ratio, Hb residual SD, and Hb CV in Hb-Var are independent factors contributing to mortality in HD patients. The inclusion of CV and abnormal ratio provides new insights into the assessment of Hb-Var, offering additional parameters for risk stratification.

The reason why we only chose to analyze all-cause mortality was that the number of patients who died of cardiovascular disease was too small to analyze cardiovascular mortality.

There are two reasons for selecting the coefficient of variation (CV) as a key parameter to reflect Hb variability. One reason is that the CV excludes differences in the mean values of patient indicators, thereby better reflecting the degree of variability in the indicators. The other reason is that, compared to parameters such as standard deviation residuals—which involve relatively complex calculations and require specialized statistical software to obtain—the CV can be calculated without specialized statistical software, making it more suitable for medical institutions in regions with limited resources. The rationale for selecting the abnormal ratio is similar to that for the CV. In addition, the calculation of abnormal ratio is simpler than CV, if our research results about it are optimistic, its popularity may better than other parameters. The practicality of these parameters enhances their potential for widespread clinical adoption.

During our analysis, we noted that 116 individuals were excluded when the subgroup was selected from the entire cohort, among whom only two were deceased—meaning that survival patients accounted for the majority of those excluded. This observation is consistent with expectation, as patients with longer survival times tend to undergo more tests, which may lead to their being classified as having a higher risk of death when analyzed as a group, due to their more detailed and comprehensive data—an indication bias. Recognizing this issue, we subsequently adjusted for the total number of tests as a covariate in the survival analysis.

It should be acknowledged that the AUC value of each parameter in our study is not very ideal, the highest value is only 0.634, and the lowest value is 0.532. Nevertheless, according to the cut-off value obtained from the ROC curve, the patient population was divided into high-risk group and low-risk group, and the results were still relatively positive. The moderate AUC values suggest that while Hb-Var parameters are significant predictors, they may be most effective when used in combination with other clinical factors.

Current research has found that, without the use of erythropoiesis-stimulating agents (ESA) for treatment intervention, hemoglobin levels in HD patients generally show a slow downward trend (4–6). The primary reasons for this are reduced renal erythropoietin production and iron metabolism abnormalities caused by chronic inflammation (18, 19). Other possible mechanisms include uremia-induced red blood cell deformity and hemolysis, folate and cobalamin deficiency, bleeding due to platelet dysfunction, and blood loss caused by hemodialysis (20, 21). The multifactorial nature of anemia in HD patients complicates its management and underscores the importance of monitoring both absolute levels and variability.

Previous studies have shown that anemia is an independent risk factor for cardiovascular disease and mortality in HD patients (22), with lower hemoglobin concentrations or hematocrit levels associated with higher mortality rates (7, 23, 24). Additionally, studies have confirmed that elevated hemoglobin levels also increase all-cause mortality in HD patients (25). At the same time, the study on the effect of ESA on the mortality of dialysis patients can also prove that stable hemoglobin concentration plays a vital role in the survival of dialysis patients. For example, Imaizumi et al.’s study demonstrated that hemoglobin levels<9.0 and ≥13.0 g/dL were significantly associated with mortality, while 10.0–10.9 g/dL levels [adjusted hazard ratio (95% CI: 1.25 (1.06–1.48) and 1.45 (1.13–1.88)), respectively]. Restricted cubic spline analysis revealed a U-shaped association between hemoglobin levels and mortality. Hemoglobin levels ≥12 g/dL were associated with mortality in patients with a history of cardiovascular disease (P interaction = 0.023) (26). While guidelines vary in their recommendations, they all agree that HD patients should maintain hemoglobin levels within a narrow, appropriate range (4, 6, 9). Regarding whether Hgb-Var is an effective predictor, previous research findings are controversial.

The observed association between higher Hb-Var and increased mortality may be explained by several pathophysiological mechanisms. Firstly, hemoglobin variability may be a surrogate marker for persistent inflammation or malnutrition-inflammation complex syndrome (MICS), which is highly prevalent in HD patients and is a strong driver of mortality (2). Inflammatory cytokines can blunt erythropoietin response and disrupt iron metabolism, leading to erratic hemoglobin levels despite ESA therapy. Secondly, variability could indicate suboptimal anemia management, such as inappropriate ESA dosing or hyporesponsiveness, which itself is linked to worse outcomes. Thirdly, large fluctuations might directly contribute to hemodynamic instability or oxidative stress, potentially exacerbating cardiovascular disease. Therefore, Hb-Var might not be a direct cause of death but rather a integrative marker of a patient’s overall clinical instability and vulnerability.

Some studies have found that hemoglobin variability is associated with increased mortality, while others have pointed out that the coefficient of variation of hemoglobin does not reflect patients’ mortality risk (15–17). Yang et al. modeled changes in hemoglobin concentration over time for each participant, then used this model to define subject’s absolute level of hemoglobin (intercept), temporal trend in hemoglobin (slope), and hemoglobin variability (residual standard deviation). The final survival analysis results showed that a 1 g/dL increase in residual standard deviation was associated with a 33% increase in mortality, even after adjusting for multiple covariates. Yang et al.’s (16) method for obtaining the residual standard deviation was similar to ours, and they also reached similar conclusions through survival analysis. However, they did not include hemoglobin variability or the proportion of outliers in their analysis.

Eckardt et al.’s (17) study used patient SD, residual SD, time-in-target, and the method of fluctuation across thresholds (target range 11.0 to 12.5 g/dL) to represent Hb variability. In their study, they also introduced a new method—combining the area under the curve (AUC) between measured Hb values and average Hb concentration—to capture the magnitude and frequency of variability as a single quantitative indicator. Multivariate-adjusted Cox regression results showed that during a median follow-up of 12.4 months, SD, residual SD, and target time AUC did not predict all-cause or CVD mortality. However, patients with persistently low Hb levels (<11 g/dL) and those whose Hb levels fluctuated between the target range and <11 g/dL had an increased risk of death. Based on this, they concluded that Hb variability is not an independent risk factor for all-cause mortality or CVD mortality (17). The reason Eckardt et al. did not obtain these results may be that their database was derived from a single private dialysis provider, EU-FME. Therefore, the generalizability to patients under the care of other providers and to countries without EU-FME centers remains unclear. Additionally, their study was conducted in Europe, while our study was conducted in China, and there may be racial differences in the relevant factors of the study. Finally, due to their use of an open-label cohort design, 44% of patients were excluded, which may have introduced selection bias.

Brunelli et al.’s (15) study represented Hgb-Var using residual standard deviation and employed logistic regression simulations based on a historically adjusted marginal structural Cox proportional hazards model. In the marginal structure analysis, the HR for Hgb-Var was 1.93. Using an unweighted model that did not address time-dependent confounding, the HR was 1.34. Based on this, they concluded that higher levels of Hgb-Var are associated with mortality in chronic HD patients.

It should be noted that Brunelli et al. (27) subsequently analyzed a smaller, later dialysis patient cohort and found no association between Hb variability and reduced survival [In the primary analysis, each g/dl increase of Hgb-Var was associated with an adjusted hazard ratio (95% confidence interval) for all-cause mortality of 1.11 (0.92 to 1.33). No significant interaction with Hgb-Var and mortality was found on the basis of age (*p* = 0.22), arterial disease (*p* = 0.45), Hgb slope (*p* = 0.68), or mean Hgb (*p* = 0.78)]. One possible explanation is that there may be important differences between the incident cohort and the prospective cohort in terms of the effects of Hb variability, or changes in anemia management over time may have led to different outcomes for Hb variability.

Our study has several inherent limitations. First, our study is a single-center study, the data originate solely from Wenzhou TCM Hospital, potentially limiting sample representativeness. Second, due to missing data in the database, we could not analyze the impact of anemia treatment (including iron supplements and ESA) on Hb-Var and mortality. Third, due to incomplete records of complications in HD patients, we were unable to analyze the impact of common complications such as type 2 diabetes and cardiovascular diseases, on their mortality. Finally, as the period of this study spanned the COVID-19 pandemic, HD patient management likely differed significantly during and after the pandemic. Despite these limitations, our study identifies Hb CV and Hb abnormal ratio as two potentially valuable predictors.

## 5 Conclusion

In conclusion, this retrospective cohort study demonstrates that hemoglobin variability, quantified by CV, residual SD, and notably the abnormal ratio, is an independent predictor of all-cause mortality in hemodialysis patients. We also identified the hemoglobin abnormal ratio as a strong and clinically intuitive predictor. Our findings suggest that beyond managing the absolute hemoglobin level, nephrologists should also pay attention to its stability over time. The CV and abnormal ratio, due to their computational simplicity and clinical interpretability, offer particularly accessible tools for risk stratification in routine practice. Future prospective, multi-center studies are warranted to validate these findings and to explore whether interventions aimed at reducing hemoglobin variability can ultimately improve survival in this vulnerable population. The implementation of these variability parameters in clinical practice could enhance risk assessment and guide personalized treatment strategies, ultimately improving outcomes for HD patients.

## Data Availability

The original contributions presented in the study are included in the article/supplementary material, further inquiries can be directed to the corresponding author.
